# A systematic approach to statistical analysis in dosimetry and patient-specific IMRT plan verification measurements

**DOI:** 10.1186/1748-717X-8-225

**Published:** 2013-09-30

**Authors:** Songbing Qin, Miao Zhang, Sung Kim, Ting Chen, Leonard H Kim, Bruce G Haffty, Ning J Yue

**Affiliations:** 1 Department of Radiation Oncology, Rutgers Cancer Institute of New Jersey, Rutgers Robert Wood Johnson Medical School, 195 Little Albany Street, 08903 New Brunswick, New Jersey, USA; 2 Department of Radiation Oncology, The First Affiliated Hospital of Soochow University, Suzhou, China

**Keywords:** Dose measurement, IMRT QA, Uncertainty, Statistical analysis

## Abstract

**Purpose:**

In the presence of random uncertainties, delivered radiation treatment doses in patient likely exhibit a statistical distribution. The expected dose and variance of this distribution are unknown and are most likely not equal to the planned value since the current treatment planning systems cannot exactly model and simulate treatment machine. Relevant clinical questions are 1) how to quantitatively estimate the expected delivered dose and extrapolate the expected dose to the treatment dose over a treatment course and 2) how to evaluate the treatment dose relative to the corresponding planned dose. This study is to present a systematic approach to address these questions and to apply this approach to patient-specific IMRT (PSIMRT) plan verifications.

**Methods:**

The expected delivered dose in patient and variance are quantitatively estimated using Student *T* distribution and *Chi* Distribution, respectively, based on pre-treatment QA measurements. Relationships between the expected dose and the delivered dose over a treatment course and between the expected dose and the planned dose are quantified with mathematical formalisms. The requirement and evaluation of the pre-treatment QA measurement results are also quantitatively related to the desired treatment accuracy and to the to-be-delivered treatment course itself. The developed methodology was applied to PSIMRT plan verification procedures for both QA result evaluation and treatment quality estimation.

**Results:**

Statistically, the pre-treatment QA measurement process was dictated not only by the corresponding plan but also by the delivered dose deviation, number of measurements, treatment fractionation, potential uncertainties during patient treatment, and desired treatment accuracy tolerance. For the PSIMRT QA procedures, in theory, more than one measurement had to be performed to evaluate whether the to-be-delivered treatment course would meet the desired dose coverage and treatment tolerance.

**Conclusion:**

By acknowledging and considering the statistical nature of multi-fractional delivery of radiation treatment, we have established a quantitative methodology to evaluate the PSIMRT QA results. Both the statistical parameters associated with the QA measurement procedure and treatment course need to be taken into account to evaluate the QA outcome and to determine whether the plan is acceptable and whether additional measures should be taken to reduce treatment uncertainties. The result from a single QA measurement without the appropriate statistical analysis can be misleading. When the required number of measurements is comparable to the planned number of fractions and the variance is unacceptably high, action must be taken to either modify the plan or adjust the beam delivery system.

## Introduction

Successful radiation treatment depends on precise calibration of the treatment machine and on the machine’s accuracy and precision in delivering that particular treatment plan. Protocols have been established to standardize the treatment machine calibration process in order to improve the accuracy of radiation dosimetry [1-5]. Similarly, various treatment quality assurance (QA) protocols and recommendations have been established and followed in many radiation treatment centers [6-10]. These protocols and recommendations typically involve dosimetric measurements, which must then be correctly interpreted in order to ensure proper radiation delivery and patient safety.

One of the major goals of radiation treatments is to deliver the desired dose coverage to the target volume. The dose coverage is determined based on dose computations by a treatment planning system (TPS) and is hopefully achieved by the radiation treatment machine. In the presence of random uncertainties arising from various components of the treatment machine, and given an infinite number of deliveries, the delivered doses would exhibit a statistical distribution with an expected variance and expected mean value. The expected mean delivered value is most likely not equal to the corresponding planned dose because current treatment planning systems do not perfectly model treatment machines. Furthermore, since a treatment course consists of a finite number of fractions, the mean value of the delivered doses over the treatment course may well differ from the expected mean value from an infinite number of fractions. Though the treatment goal is often stated simply as a desired dose (planned dose) delivered to a patient, it would be more accurate to state the goal as delivering a mean dose over a treatment course to a patient within a certain confidence interval (e.g., 95% confidence interval of 3%) around the desired dose. It has been generally accepted that delivered dose to patient should be within 5% of desired one with a 95% confidence level [11,12], and its precision is affected by uncertainties in every step of radiation treatment process. The goal of this paper is to focus on the radiation delivery step and to present an approach to estimate and evaluate whether to-be-delivered doses over a treatment course meet the treatment goal during the radiation delivery.

For more complicated treatment deliveries such as IMRT, patient-specific IMRT (PSIMRT) plan verification QA is usually performed. The purpose of the PSIMRT is to verify the computed dose distribution of a plan is accurate by conducting measurements in (typically) homogeneous phantoms. If the PSIMRT result is deemed acceptable according to certain criteria, the implicit assumption is that the plan, when delivered to patient via a same delivery system, will deliver similarly acceptable doses. The ultimate aim of PSIMRT QA is to ensure plan integrity at treatment and agreement (within a certain tolerance) between delivered and planned dose over a course of treatment. In PSIMRT QA, even after the integrity of plan transfer and treatment machine performance is thoroughly inspected and verified, the existence of measurement uncertainties is well-documented [13,14], and investigations have been conducted to incorporate those uncertainties into IMRT treatment delivery and planning [15,16]. With these uncertainties, it is almost certain that repeated PSIMRT QA measurements will produce a statistical distribution. Therefore, it may be inappropriate to draw a conclusion from PSIMRT QA based on a single measurement. For example, under conditions of correct plan transfer, normal machine function, and proper measurement equipment, it is a relatively common scenario where an initial PSIMRT QA measurement fails to meet pre-set criteria [17,18], then subsequently passes on repeat measurement, and a decision must be made whether the plan is acceptable or not. Obviously, according to the general statistical theories, the final decision should not completely ignore the initial failure even if subsequent measurements are acceptable. The decision should be based on statistical analysis of the QA measurements including both the failed and passing results and the expected treatment goal. Furthermore, it is intuitive that a treatment course of fewer fractions requires a higher standard in the distribution of its QA results, e.g., for stereotactic body radiotherapy (SBRT). Therefore, the ultimate goal of PSIMRT QA should be three-fold: (1) and most importantly to verify the integrity of plan transfer from the TPS to the treatment unit, to identify major discrepancy such as beam modeling errors, and accelerator/MLC performance, (2) to check the deliverability of the plan, and (3) to evaluate the variation of the plan delivery would be within the statistical tolerance of the treatment prescription based on the fractionation scheme and the measured variation.

In general, there are two types of errors: systematic errors and random errors. Systematic errors are normally caused by inaccuracy in a system or a tendency to consistently be off from a predicted value. Random errors are unpredictable, unknown, and fluctuating variations. Several studies have demonstrated that a finite number of fractions lead to residual errors in total doses delivered to patient [19-21]. According to statistical theory, for a dose quantity of random errors, the expected value and expected standard deviation (SD) of its statistical distribution are unknown but can be estimated using results from a number of repeated and independent measurements. In many cases, the measured mean and standard deviation are directly used as the expected value and expected standard deviation, respectively. However, substitution of a measured mean value for an expected value is scientifically meaningful only if a confidence interval and its corresponding confidence level for the substitution are clearly specified. The confidence interval and confidence level, in turn, are highly dependent on the number and variance of measurements. With the expected value and expected SD of the dose quantity statistically determined, based on the "finite-sample distribution theory" of a given statistics, the mean value distribution of the dose quantity delivered over a limited number of fractions can be statistically estimated and is highly dependent on the number of fractions.

Therefore, theoretically, the evaluation of PSIMRT plan verification QA results (and other similar dosimetric measurement procedures) should not be based on a preselected value or a single observation of pass/fail. Rather it should be based on a statistical approach incorporating the number and variance of measured results, the associated accuracy confidence interval and level, and related treatment details such as number of fractions, uncertainties during treatment, and desired dosimetric tolerance. Additionally, uncertainties exist in the measurement equipment and measurement setups. These uncertainties should also be carefully analyzed and taken into consideration for the evaluation of measurement results.

The current study is attempted to build a closed and complete statistical model and expand the scope of the newly improved statistical model and method to include another realm where such a method would be beneficial: dosimetry and PSIMRT plan verification QA measurements

## Materials and methods

In the subsequent sections, unless otherwise stated, the term dose or dose value is referred to the dose at a specific point in patient or phantom.

For clarification, a few notations are first defined:

• *PD*_*X*_: Percent Difference between the expected value and mean value of subject *X*;

• *P*: Probability of the subject of interest;

• *N*: Number of fractions of a treatment course

• *n*: Number of pre-treatment QA measurements

• *R*: the expected dose value at a point in QA measurement

• *σ*^*2*^: the expected dose variance at a point in QA measurement

• ${\bar{R}}_{n}$: the mean of measured results for dose at a point from *n* pre-treatment QA measurements

• ${\bar{\sigma }}_{n}^{2}$: the variance of measured results for dose at a point from *n* pre-treatment QA measurements

• ${\bar{R}}_{N}$: the mean dose quantity at a point delivered over *N* fractions of treatment

• $\sigma_{N}^{2}$: the variance of the mean dose quantity at a point delivered over *N* fractions of treatment

For complicated treatments and plans, such as those involving IMRT and requiring higher delivery accuracy, the questions are 1) how to estimate to-be-delivered dose over a treatment course and its deviation from the planned dose, 2) how to use pre-treatment QA measurements to infer these dose estimates for the to-be-delivered treatment and evaluate the QA outcome accordingly.

Assuming all machine components and the plan transfer are within specification, in a typical PSIMRT plan verification QA procedure, the current practice is to conduct measurement and compare the measured result to the corresponding planned value, typically in a homogeneous phantom. If the difference is smaller than certain preset criteria, the IMRT treatment design is deemed acceptable. As discussed above, the delivered radiation dose most likely exhibits a statistical distribution if repeated, even using the same IMRT plan and radiation treatment machine. Furthermore, the average dose and its deviation over a treatment course also present a statistical distribution and vary with the number of treatment fractions. Thus, a more scientific approach to PSIMRT plan verification QA procedures is not to simply compare the QA results with the corresponding plan value(s) but to adopt a systematic approach to conduct statistical analysis on the QA results, taking into account treatment details such as the number of fractions and desired deviation tolerance.

### General formalism for measurement statistical analysis

Assuming a dose quantity has uncertainties (random) and its value follows a certain statistical distribution if it is delivered an infinite number of times, there exist an expected value *R* and an expected variance *σ*^*2*^ for this dose quantity. There are two types of statistical estimations for this dose quantity: 1) estimation of the expected *R* and *σ*^*2*^ by conducting *n* measurements and 2) estimation of the mean value and variance of the dose quantity when it is to be delivered for a limited number *N* times. While pre-treatment measurements of a dose quantity fall into the first type, the second type is analogous to the estimation of the dose delivered to a patient over a treatment course. In this study, it is assumed that the statistical distribution of a dose quantity follows a Normal Distribution.

#### Estimation of the percent difference between the expected and QA measured results

Statistically, the expected value *R* is not exactly known for almost all cases. To determine the expected value, *n* independent measurements are conducted with results of *R*_*1*_*, R*_*2*_*, …,* and *R*_*n*_, respectively. The quantity

(1) $$T=\frac{{\bar{R}}_{n}-R}{{\bar{\sigma }}_{n}/\sqrt{n}}$$

follows the "Student *T* Distribution" with (n-1) degrees of freedom and has the probability density function (16)

(2) $$f\left(t\right)=\frac{\Gamma \left(n/2\right)}{\sqrt{\pi \left(n-1\right)}\Gamma \left(\left(n-1\right)/2\right)}{\left(1+\frac{t^{2}}{n-1}\right)}^{-n/2}$$

where ${\bar{R}}_{n}=\frac{\sum_{i=1}^{n}R_{i}}{n}$ and ${\bar{\sigma }}_{n}^{2}=\frac{\sum_{i=1}^{n}{\left(R_{i}-{\bar{R}}_{n}\right)}^{2}}{n-1}$ are the mean value and the variance of the *n* measurement results, respectively, and *Γ(x)* is the Gamma function and can be expressed as $\Gamma \left(x\right)=\int_{0}^{\infty }y^{x-1}e^{-y}\mathrm{dy}$. It should be noted that the expected variance *σ*^*2*^ is different from ${\bar{\sigma }}_{n}^{2}$. Whereas ${\bar{\sigma }}_{n}^{2}$ is a measured quantity, the expected variance $\sigma^{2}=\underset{n\to \infty }{\lim}{\bar{\sigma }}^{2}$.

The probability of quantity *T* satisfying condition *a* ≤ *T* ≤ *b* is computed as

(3) $$P\left(a\leq T\leq b\right)=\int_{a}^{b}f\left(t\right)\mathrm{dt}$$

By combining equations (1) through (3), it is obtained that

(4) $$\begin{array}{ll} \;P\left(a\leq \frac{R-{\bar{R}}_{n}}{{\bar{\sigma }}_{n}/\sqrt{n}}\leq b\right) & =P\left(\frac{a{\bar{\sigma }}_{n}/\sqrt{n}}{{\bar{R}}_{n}}100\%\leq \frac{R-{\bar{R}}_{n}}{{\bar{R}}_{n}}100\%\leq \frac{b{\bar{\sigma }}_{n}/\sqrt{n}}{{\bar{R}}_{n}}100\%\right)\\ & =\int_{a}^{b}\frac{\Gamma \left(n/2\right)}{\sqrt{\pi \left(n-1\right)}\Gamma \left(\left(n-1\right)/2\right)}{\left(1+\frac{t^{2}}{n-1}\right)}^{-n/2}\mathrm{dt} \end{array}$$

In Eq. 4, $\frac{R-{\bar{R}}_{n}}{{\bar{R}}_{n}}100\%$, denoted as *PD*_*R*_, is the percent difference between the expected result for the procedure under consideration and the mean value of the measurement results. From the equation, it is apparent that the probability distribution of *PD*_*R*_ is independent of the expected variance *σ*^*2*^ and can be determined with the parameters of a particular measurement process, such as the number, the mean and variance of the measured results.

From Eqn. 4, the probability of |*PD*_*R*_| ≤ *y%*, *y* ≥ 0 (the probability of the measurement accuracy being within *y%*) can be determined as

(5) $$\begin{array}{l} P\left(\left|PD_{R}\right|\leq \frac{A{\bar{\sigma }}_{n}/\sqrt{n}}{{\bar{R}}_{n}}100\%=\mathrm{y\%}\right)\\ \;=\int_{-A}^{A}\frac{\Gamma \left(n/2\right)}{\sqrt{\pi \left(n-1\right)}\Gamma \left(\left(n-1\right)/2\right)}{\left(1+\frac{t^{2}}{n-1}\right)}^{-n/2}\mathrm{dt} \end{array}$$

where $A=\frac{{\bar{R}}_{n}\sqrt{n}}{{\bar{\sigma }}_{n}}\times \mathrm{y\%}$.

Equation (5) indicates that the probability of measurement accuracy is dependent on the number (*n*), mean (${\bar{R}}_{n}$) and deviation (${\bar{\sigma }}_{n}$) of the measurement results and can be computed directly from the measurement process itself.

It should be emphasized that the expected value is unknown unless there exists zero uncertainty. What are known from a measurement procedure are the measurement results and their distribution.

#### Estimation of the expected standard deviation

The quantity $Q=\frac{{\bar{\sigma }}_{n}\sqrt{\left(n-1\right)}}{\sigma }$ follows "*Chi* Distribution" with n-1 degrees of freedom, with a probability density of $f_{Q}\left(t\right)=\frac{t^{\left(n-1\right)-1}e^{-t^{2}/2}}{2^{\frac{n-1}{2}-1}\Gamma \left(\frac{n-1}{2}\right)}$ and *t* ≥ 0.

There exists relationship $\sigma =E\left({\bar{\sigma }}_{n}\right)\frac{\Gamma \left(\frac{n-1}{2}\right)}{\Gamma \left(\frac{n}{2}\right)}\sqrt{\frac{n-1}{2}}$, where $E\left({\bar{\sigma }}_{n}\right)$ is the expected value of ${\bar{\sigma }}_{n}$. *σ* can be estimated with term ${\bar{\sigma }}_{n}\frac{\Gamma \left(\frac{n-1}{2}\right)}{\Gamma \left(\frac{n}{2}\right)}\sqrt{\frac{n-1}{2}}$.

#### Probability determination of the percent difference between the expected measurement value and a given value

As stated previously, the dose from the treatment plan (given value) is most likely not equal to the expected dose delivered by the treatment machine.

Suppose a given value is *R*_*g*iven_ and the expected measured value is *R*. Equation (4) can be rewritten as

(6) $$\begin{array}{l} P\left(\frac{a{\bar{\sigma }}_{n}/\sqrt{n}}{{\bar{R}}_{n}}100\%\leq \frac{R-{\bar{R}}_{n}}{{\bar{R}}_{n}}100\%\leq \frac{b{\bar{\sigma }}_{n}/\sqrt{n}}{{\bar{R}}_{n}}100\%\right)\\ \;=P\left(\frac{a{\bar{\sigma }}_{n}/\sqrt{n}+{\bar{R}}_{n}-R_{\mathrm{given}}}{R_{\mathrm{given}}}100\%\leq \frac{R-R_{\mathrm{given}}}{R_{\mathrm{given}}}100\%\right)\\ \;\left(=PD_{V}\leq \frac{b{\bar{\sigma }}_{n}/\sqrt{n}+{\bar{R}}_{n}-R_{\mathrm{given}}}{R_{\mathrm{given}}}100\%\right)\\ \;=\int_{a}^{b}\frac{\Gamma \left(n/2\right)}{\sqrt{\pi \left(n-1\right)}\Gamma \left(\left(n-1\right)/2\right)}{\left(1+\frac{t^{2}}{n-1}\right)}^{-n/2}\mathrm{dt} \end{array}$$

where *a* and *b* are two values to be determined.

The probability for $\left|\frac{R-R_{\mathrm{given}}}{R_{\mathrm{given}}}100\%\right|\leq \mathrm{x\%}$ (*x* ≥ 0) is then

(7) $$\begin{array}{ll} \;P & \left(\left|\frac{R-{\bar{R}}_{n}}{{\bar{R}}_{n}}100\%\right|\leq \mathrm{x\%}\right)\\ & =P\left(\frac{a{\bar{\sigma }}_{n}/\sqrt{n}+{\bar{R}}_{n}-R_{\mathrm{given}}}{R_{\mathrm{given}}}100\%=-\mathrm{x\%}\leq \frac{R-R_{\mathrm{given}}}{R_{\mathrm{given}}}100\%\right)\\ & \;\left(\leq \frac{b{\bar{\sigma }}_{n}/\sqrt{n}+{\bar{R}}_{n}-R_{\mathrm{given}}}{R_{\mathrm{given}}}100\%=\mathrm{x\%}\right)\\ & =\int_{\frac{\sqrt{n}}{{\bar{\sigma }}_{n}}\left(R_{\mathrm{given}}-{\bar{R}}_{n}-\mathrm{x\%}R_{\mathrm{given}}\right)}^{\frac{\sqrt{n}}{{\bar{\sigma }}_{n}}\left(R_{\mathrm{given}}-{\bar{R}}_{n}+\mathrm{x\%}R_{\mathrm{given}}\right)}\frac{\Gamma \left(n/2\right)}{\sqrt{\pi \left(n-1\right)}\Gamma \left(\left(n-1\right)/2\right)}\\ & \;\times {\left(1+\frac{t^{2}}{n-1}\right)}^{-n/2}\mathrm{dt} \end{array}$$

Assuming $R_{\mathrm{Given}}=\left(1+\Delta \right){\bar{R}}_{n}$ where *Δ* can be either positive or negative, Equations 6 and 7 become

(8) $$\begin{array}{ll} \;P & \left(\left|\frac{R-R_{\mathrm{given}}}{R_{\mathrm{given}}}100\%\right|\leq \mathrm{x\%}\right)\\ & =\int_{\frac{{\bar{R}}_{n}\sqrt{n}}{{\bar{\sigma }}_{n}}\left[\Delta -\mathrm{x\%}\left(1+\Delta \right)\right]}^{\frac{{\bar{R}}_{n}\sqrt{n}}{{\bar{\sigma }}_{n}}\left[\Delta +\mathrm{x\%}\left(1+\Delta \right)\right]}\frac{\Gamma \left(n/2\right)}{\sqrt{\pi \left(n-1\right)}\Gamma \left(\left(n-1\right)/2\right)}{\left(1+\frac{t^{2}}{n-1}\right)}^{-n/2}\mathrm{dt} \end{array}$$

#### Estimation of the dose delivered to a patient over a treatment course

Assume that the dose uncertain variance in a fraction of radiation treatment is $\sigma^{2}=\sigma_{\mathrm{other}}^{2}+\sigma_{m}^{2}$, where $\sigma_{m}^{2}$ is the uncertain variance originated from radiation delivery machine and $\sigma_{\mathrm{other}}^{2}$ is that from all other sources including patient setup, organ motion, etc. For a treatment course of *N* fractions, the mean dose ${\bar{R}}_{N}$ over the course follows a normal distribution with the expected value *R* and a variance $\sigma_{N}^{2}=\frac{\sigma^{2}}{N}$:

(9) $$P\left(a\leq {\bar{R}}_{N}\leq b\right)=\int_{a}^{b}\frac{\sqrt{N}}{\sigma \sqrt{2\pi }}e^{-\frac{N\left(t-R\right)}{2\sigma^{2}}}\mathrm{dt}$$

It can be inferred from Eqn. 9 that for a treatment course with a large number of fractions the mean value of a dose quantity delivered to the patient is likely to be close to the expected value even if the dose delivery uncertainty of one fraction (variance *σ*) is large. However, for a treatment course of few fractions, such as SBRT treatment, dose delivery uncertainty must be reduced to ensure that dose delivered to patient is close to the expected value.

For example, suppose that the mean dose delivered to a patient over a treatment course is required to be within 3% of the expected value with a 95% confidence level, then $2\sigma_{N}=\frac{2\sigma }{\sqrt{N}}=3\%$ and $\sigma =1.5\%\times \sqrt{N}$. If the number of fractions is 30, the treatment quality can be maintained as long as the delivery uncertainty (standard deviation) is within 8.2%. However, if the number of fractions is 3 as in many SBRT treatments, the delivery uncertainty must be reduced to 2.6% to ensure treatment quality. Assuming that the uncertainty *σ*_*others*_ from all other sources is 2%, the machine delivery uncertainty should be controlled below $\sqrt{{\left(8.2\%\right)}^{2}-{\left(2\%\right)}^{2}}=7.6\%$ to achieve the 3% criteria for the 30 fractions of treatment; while for a SBRT treatment of 3 fractions, the machine delivery uncertainty must be controlled below $\sqrt{{\left(2.6\%\right)}^{2}-{\left(2\%\right)}^{2}}=1.7\%$, a much higher requirement than the conventional treatment of 30 fractions. Apparently, if *σ*_*others*_ is higher than 2.6%, there is no way to achieve the expected treatment accuracy for the SBRT treatments and measures have to be taken to reduce *σ*_*others*_.

Eqn. 9 can be further expanded to infer the probability of difference between a given dose value and the mean dose over a course of treatment from the pre-treatment QA results as follows. Assuming ${\bar{R}}_{N}=R_{\mathrm{Given}}\left(1+\delta \%\right)$, where *δ* can be either positive or negative,

$$\begin{array}{ll} \;P & \left(a-R_{\mathrm{Given}}\leq {\bar{R}}_{N}-R_{\mathrm{Given}}\leq b-R_{\mathrm{Given}}\right)\\ & =P\left(\frac{a}{R_{\mathrm{Given}}}-1\leq \delta \%\leq \frac{b}{R_{\mathrm{Given}}}-1\right)=\int_{a}^{b}\frac{\sqrt{N}}{\sigma \sqrt{2\pi }}e^{-\frac{N\left(t-R\right)}{2\sigma^{2}}}\mathrm{dt} \end{array}$$

where *δ* is the percent difference between a given dose value (e.g., planned dose value ) and the expected mean dose value delivered over *N* fractions. *δ* is different from *Δ* which is the percent difference between a given dose value and the expected dose value in a single delivery.

Averaging over all possible values of *R* and σ using equations and terms in sections a) and b),

(10) $$\begin{array}{l} <P\left(\left|\delta \%\right|\leq \mathrm{z\%}\right){>}_{R,\sigma }=\int_{\left(1-\mathrm{z\%}\right)R_{\mathrm{Given}}}^{\left(1+\mathrm{z\%}\right)R_{\mathrm{Given}}}<\frac{\sqrt{N}}{\sigma \sqrt{2\pi }}e^{-\frac{N\left(t-R\right)}{2\sigma^{2}}}{>}_{R,\sigma }\mathrm{dt}\\ =\int_{\left(1-\mathrm{z\%}\right)R_{\mathrm{Given}}}^{\left(1+\mathrm{z\%}\right)R_{\mathrm{Given}}}\mathrm{dt}\int_{-\infty }^{+\infty }\frac{-{\left(\frac{{\bar{\sigma }}_{n}\sqrt{\left(n-1\right)}}{\sqrt{\sigma_{\mathrm{other}}^{2}+\sigma_{m}^{2}}}\right)}^{\left(n-1\right)}e^{-{\left(\frac{{\bar{\sigma }}_{n}\sqrt{\left(n-1\right)}}{\sqrt{\sigma_{\mathrm{other}}^{2}+\sigma_{m}^{2}}}\right)}^{2}/2}}{2^{\frac{n-1}{2}-1}\Gamma \left(\frac{n-1}{2}\right)\sqrt{\sigma_{\mathrm{other}}^{2}+\sigma_{m}^{2}}}\\ \;\times F\left(t\right)d\sigma_{m} \end{array}$$

Where $F\left(t\right)=\int_{-\infty }^{+\infty }\frac{\sqrt{N}}{\sqrt{\sigma_{\mathrm{other}}^{2}+\sigma_{m}^{2}}\sqrt{2\pi }}e^{-\frac{N\left(t-R\right)}{2\sigma^{2}}}\frac{-\Gamma \left(n/2\right)\frac{{\bar{\sigma }}_{n}}{\sqrt{n}}\mathrm{dR}}{\sqrt{\pi \left(n-1\right)}\Gamma \left(\left(n-1\right)/2\right)}{\left(1+\frac{{\left(\frac{{\bar{R}}_{n}-R}{{\bar{\sigma }}_{n}/\sqrt{n}}\right)}^{2}}{n-1}\right)}^{-n/2}$and where *z%* ≥ 0 and is desired accuracy to the given value.

Eqn. 10 establishes the statistical relationship for the pre-treatment QA measurement results (*n*, ${\bar{R}}_{n}$ and ${\bar{\sigma }}_{n}$), the treatment mean dose from to-be-delivered treatment course of *N* fractions, and the difference between the treatment mean dose and a given (planned) dose value *R*_*given*_. In the cases where *R* and σ_m_ are evaluated to be around ${\bar{R}}_{n}$ and $E\left({\bar{\sigma }}_{n}\right)$ within a very small interval of high confidence level, ${\bar{R}}_{n}$ and $E\left({\bar{\sigma }}_{n}\right)$ can be used to directly substitute *R* and σ_m_ in Eqn. 10, respectively, to simplify the mathematical computation. In this case, Eqn. 10 becomes

(11) $$\begin{array}{l} <P\left(\left|\delta \%\right|\leq \mathrm{z\%}\right){>}_{R,\sigma }\\ \;=\int_{\left(1-\mathrm{z\%}\right)R_{\mathrm{Given}}}^{\left(1+\mathrm{z\%}\right)R_{\mathrm{Given}}}\frac{\sqrt{N}}{\sqrt{\sigma_{\mathrm{other}}^{2}+\sigma_{m}^{2}}\sqrt{2\pi }}e^{-\frac{N\left(t-\bar{R}\right)}{2\left(\sigma_{\mathrm{other}}^{2}+\sigma_{m}^{2}\right)}}\mathrm{dt} \end{array}$$

where

(12) $$\sigma_{m}\approx {\bar{\sigma }}_{n}\frac{\Gamma \left(\frac{n-1}{2}\right)}{\Gamma \left(\frac{n}{2}\right)}\sqrt{\frac{n-1}{2}}$$

Using the formalisms presented in sections a) through d), probabilities can be quantitatively derived for relationships among the expected measurement value, a given value, and the expected treatment mean dose over a treatment course using the information readily available from the QA measurement procedure, assuming there is not additional patient treatment uncertainties. However, in clinical treatments, the value of *σ*_*others*_ needs to be carefully estimated based on various factors such as immobilization devices, IGRT devices, etc.

### Application of the above-derived analytical method to dosimetric and clinical PSIMRT plan verification QA measurements

As a demonstration, the above derived equations were used to analyze a clinical PSIMRT plan verification QA procedure and to predict the expected treatment mean dose over a treatment course. The QA measurements were carried out using four 0.05 cc Exradin Model A1SL ionization chambers and Standard Imaging 8 channel TomoElectrometer (Standard Imaging, Middleton, WI) on a Tomothearpy Hi-Art™ machine (Tomotherapy-Accuray, Madison, WI). The four chambers were positioned inside a "Cheese Phantom" (Tomotherapy-Accuray, Madison, WI) at four locations of various dose gradients. The measurements were repeated multiple times. Each of the measurements was independent (meaning that the measurements did not affect each other), and the equipment was identical for each measurement. Temperature and pressure were corrected for all the measurements.

The variance in a QA measurement procedure is attributed to the variances of both machine delivery and measurement instruments. However, during a treatment course the variance of measurement instruments are absent while patient setup uncertainty can also contribute to the treatment variance. To simulate the patient setup variance to a limited extent, some of the QA measurements presented in the following section were conducted by re-setting the phantom position and then taking measurements. Certainly, this simulation could underestimate the degrees of uncertainty in patient setup since unlike phantom patient body is generally not rigid and may also experience intra-fractional motion.

## Results

The results presented in this study were measured after careful examinations of the machine and measurement instruments to ensure they functioned normally and within specification. If large deviations in the results were observed, investigations were also conducted on the machines and instruments to verify their working integrity.

### Statistical analysis of the accuracy of dose QA measurement results

Table 1a and 1b present the results and statistical analysis of the dose measurement procedure for two of the four ionization chambers. The results from the other two chambers were very similar to those of Chamber 1 and are not presented. The doses from the plan were 1.702 and 0.546 Gy, respectively, at the corresponding points of Chambers 1 and 2. The measurements were conducted independently after initially setting up the phantom and without resetting up the phantom between each measurement.

**Table 1:** Patient specific IMRT QA absolute dose measurement analysis

**Meas. Num (n)**	**Measurement result (Gy)**	**Mean value (1 to n)**	**Result standard deviation (1 to n)**	**Estimated dose delivery standard deviation**	**Probability for the mean to be within 2% accuracy (1 to n)**	**Probability for the mean to be within 3% accuracy (1 to n)**
**a.** Chamber 1: corresponding plan dose was 1.702 Gy						
1	1.706	1.706	-		-	-
2	1.695	1.700	0.008	0.010	89.63%	93.05%
3	1.695	1.699	0.006	0.007	98.85%	99.50%
4	1.689	1.696	0.007	0.008	99.76%	99.93%
5	1.689	1.695	0.007	0.007	99.96%	99.98%
**b.** Chamber 2: corresponding plan dose was 0.546 Gy						
1	0.513	0.513	-		-	-
2	0.507	0.510	0.005	0.006	80.50%	86.76%
3	0.504	0.508	0.005	0.006	93.11%	96.77%
4	0.504	0.507	0.005	0.005	97.94%	99.33%
5	0.502	0.506	0.005	0.005	99.25%	99.83%

The measurements were conducted without the QA phantom re-setup between each measurement and the statistical analysis was for the accuracy of measurement itself. The accuracy is defined as percent difference between the expected value and measurement mean value.

The statistical results were presented for the measurement accuracy analysis. The measurement accuracy was defined as the percent difference between the expected dose value (not the plan value) and the measurement mean value. The analysis estimated the expected dose delivered from the machine assuming that the measurement instruments had minimal uncertainties.

In the tables, columns 1 to 7 are, respectively, the measurement sequence number n (n =1, 2, … , 6), the measurement raw data value *R*_*n*_, the mean value ${\bar{R}}_{n}=\frac{\sum_{i=1}^{n}R_{n}}{n}$, the standard deviation ${\bar{\sigma }}_{n}=\sqrt{\frac{\sum_{i=1}^{n}{\left(R_{i}-{\bar{R}}_{n}\right)}^{2}}{n-1}}$, the estimated expected dose delivery standard deviation, the probability *P*_*n*_(*PD*_*R*_ ≤ 2*%*), and the probability *P*_*n*_(*PD*_*R*_ ≤ 3*%*) computed from Eqn. 5. *P*_*n*_(*PD*_*R*_ ≤ 2*%*) and *P*_*n*_(*PD*_*R*_ ≤ 3*%*) were the probabilities of the measurement accuracy being better than 2% and 3%, respectively, if their corresponding mean values were used for the expected value after *n* measurements.

Using the measurement results from Chamber 1 as an example, the systematic analysis for this particular measurement process can be described as follows. According to Eqns. 1–4 and intuitively, it is improbable to evaluate the accuracy using just the very first measurement reading 1.706 Gy. After the second reading was taken (1.695 Gy), the mean value of the two readings was 1.700 Gy. Using Eqn. 5, the probability of percent difference between this mean value and the expected measurement value being less than 2% was 89.63%. In other words, there was 89.63% probability that the accuracy of this mean value was better than 2%. With a third measurement (1.695), that probability (or confidence level) increased to 98.85%. As more readings were taken, this probability increased. Assuming that the accuracy requirement for this particular measurement process was 2% and confidence level requirement was 95%, it is apparent that in this particular case 3 measurements had to be conducted to achieve the confidence level to substitute the measured mean value for the expected value. However, if the required confidence level was still 95% but the required accuracy was 3%, 2 total measurements, instead of 3, would have been adequate (column 7 of Table 1).

Similar analysis can be performed for Chamber 2 measurements.

Table 2a and 2b present similar results for Chambers 1 and 2 in a little different measurement design. Instead of leaving phantom untouched between each measurement, the phantom, along with the chambers (which were embedded inside the phantom) was reset up and realigned between each measurement. This measurement design was intended, to a limited degree, to simulate the setup uncertainty. As expected, the standard deviations of the corresponding results were larger. After 5 measurements, the confidence levels to achieve an accuracy of 3% were 83.87% and 82.01%, respectively, for Chambers 1 and 2 measurements; and were 95.41% and 94.62%, respectively, for an accuracy of 5%. If the setup uncertainty was taken into account, 5 measurements were still inadequate to derive a mean value with which the expected dose value was within 3% of the mean value at 95% confidence level; however, the 5 measurements were sufficient if the accuracy requirement was 5% at the confidence level of 95%.

**Table 2:** Patient specific IMRT QA absolute dose measurement analysis

**Meas. num (n)**	**Measurement result (Gy)**	**Mean value (1 to n)**	**Result standard deviation (1 to n)**	**Estimated dose delivery standard deviation**	**Probability for the mean to be within 3% accuracy (1 to n)**	**Probability for the mean to be within 5% accuracy (1 to n)**
**a.** Chamber 1: corresponding plan dose was 1.702 Gy						
1	1.689	1.689	-		-	-
2	1.706	1.698	0.012	0.015	89.62%	93.73%
3	1.550	1.648	0.086	0.097	57.74%	76.25%
4	1.700	1.661	0.075	0.081	72.60%	88.76%
5	1.678	1.665	0.065	0.069	83.87%	95.41%
**b.** Chamber 2: corresponding plan dose was 0.546 Gy						
1	0.502	0.502	-		-	-
2	0.466	0.484	0.025	0.032	43.55%	59.61%
3	0.457	0.475	0.024	0.027	59.52%	77.70%
4	0.465	0.473	0.020	0.022	75.01%	90.16%
5	0.453	0.469	0.019	0.021	82.01%	94.62%

The measurements were conducted with the QA phantom re-setup and re-aligned between each measurement and the statistical analysis was for the accuracy of measurement itself. The accuracy is defined as percent difference between the expected value and measurement mean value.

### Statistical analysis of the differences between measurement dose and corresponding plan dose

Following Equations 6, 7 an 8, Table 3 present the statistical analysis results for the percent differences between the plan doses and the expected doses delivered from the machine at the locations of Chambers 1 and 2. The results in Table 3a and 3b correspond to the measurements without the phantom reset up, while those in Table 3c and 3d were with the phantom being reset up and realigned.

**Table 3:** Patient specific IMRT QA absolute dose measurement analysis

**Meas. num (n)**	**Percent difference between the mean and plan dose (%, 1 to n)**	**Probability for the mean to be within 3% of plan value (1 to n)**	**Probability for the mean to be within 5% of plan value (1 to n)**
**a.** Chamber 1: corresponding plan dose was 1.702 Gy. No QA phantom re-setup between each measurement.			
1	0.232	-	-
2	-0.095	93.05%	95.81%
3	-0.205	99.49%	99.84%
4	-0.341	99.93%	99.97%
5	-0.423	99.98%	99.98%
**b.** Chamber 2: corresponding plan dose was 0.546 Gy. No QA phantom re-setup between each measurement.			
1	-5.960	-	-
2	-6.551	3.28%	9.96%
3	-6.939	0.70%	3.12%
4	-7.132	0.10%	0.70%
5	-7.319	0.02%	0.17%
**c.** Chamber 1: corresponding plan dose was 1.702 Gy. The QA phantom was re-setup between each measurement.			
1	-0.751	-	-
2	-0.259	89.57%	93.72%
3	-3.153	39.74%	65.16%
4	-2.388	55.53%	81.86%
5	-2.193	65.01%	90.52%
**d.** Chamber 2: corresponding plan dose was 0.546 Gy. The QA phantom was re-setup between each measurement.			
1	-8.065	-	-
2	-11.327	4.76%	8.88%
3	-12.963	1.69%	3.35%
4	-13.432	0.39%	0.85%
5	-14.164	0.07%	0.18%

Statistical analysis results for the percent difference between the expected delivery value and given (plan) value.

It should be re-emphasized that the plan dose is not at all necessarily equal to the expected dose delivered from the machine.

Without taking the phantom re-setup into account (Table 3a), at the location of Chamber 1, two measurements were adequate to have high confidence (95.81%) that the expected machine delivery dose would be within 5% of the corresponding plan dose; after three measurements, one would have high confidence (99.49%) that it would be within 3% of the plan dose. However, with the uncertainty caused by the phantom re-setup taken into account, although the mean value of the five measurements was well within 3% (-2.2%) of the plan dose, the confidence level (65.01%) was fairly low for the expected delivery dose being within 3% of the plan dose. The confidence level was only 90.52% for the expected delivery dose to be within 5% of the plan dose after 5 measurements.

These results demonstrate that simple comparison of measured mean value to a given value is insufficient to draw a statistically meaningful conclusion about the difference between the expected measurement value and the given value. Appropriate statistical analysis has to be conducted.

As shown in Table 3b and 3d, it was statistically impossible for the expected delivery dose at the location of Chamber 2 to be within 5% of the corresponding plan dose. A different physical quantity such as Gamma Index [22] needs to be used for the QA outcome evaluation.

### Statistical estimation of the difference between plan dose and the average dose delivered over a treatment course

Table 4 show the results of statistical estimation of the differences between plan dose and the average dose delivered over a treatment course at the locations of Chambers 1 and 2. These results could be used to estimate the difference between plan dose and the corresponding dose delivered to patient over a treatment course. The results were derived from Eqn. 11 using the QA measurement data. Two types of treatment course were analyzed: one with 36 fractions, similar to a conventional treatment course; the other with 3 fractions, similar to the fractionation scheme in a SBRT treatment course. Similarly, Table 4a,b,c and d are correspondent to the measurements without and with the phantom re-setup, respectively. In Table 4e and f, on top of the QA measurement uncertainties introduced from dose delivery and phantom re-setup, an additional 5% random uncertainty was added. This 5% of uncertainty was estimated to be caused by other factors during patient treatment, such as organ motion, body contour change, clinical patient setup, etc. The overall standard deviations in Table 4e and f were computed as $\sigma_{\mathrm{All}}=\sqrt{\sigma_{\mathrm{measurement}}^{2}+{\left(5\%\times \bar{R}\right)}^{2}}$ (13) and used in Eqn. 12.

**Table 4:** Statistical estimation of the difference between planned dose and the average dose delivered over a treatment course

**Number of fractions (N)**	**Probability for the mean to be within 1% of plan value**	**Probability for the mean to be within 3% of plan value**	**Probability for the mean to be within 5% of plan value**
**a.** Estimation at the point corresponding to Chamber 1: corresponding plan dose was 1.702 Gy. No QA phantom re-setup between **each measurement. The mean measured value used was 1.684 Gy.**			
36	20.23%	100.00%	100.00%
3	40.59%	100.00%	100.00%
**b**. Estimation at the point corresponding to Chamber 2: corresponding plan dose was 0.546 Gy. No QA phantom re-setup between each measurement. The mean measured value used was 0.503 Gy.			
36	0.00%	0.00%	0.00%
3	0.00%	0.00%	0.00%
**c.** Estimation at the point corresponding to Chamber 1: corresponding plan dose was 1.702 Gy. The QA phantom was re-setup between each measurement. The mean measured value used was 1.669 Gy.			
36	8.21%	94.12%	100.00%
3	23.89%	65.58%	90.16%
**d.** Estimation at the point corresponding to Chamber 2: corresponding plan dose was 0.546 Gy. The QA phantom was re-setup between each measurement. The mean measured value used was 0.471 Gy.			
36	0.00%	0.00%	0.00%
3	0.00%	0.00%	0.00%
**e.** Estimation at the point corresponding to Chamber 1: corresponding plan dose was 1.702 Gy. The QA phantom was re-setup between eachmeasurement and additional 5% uncertainty was included. The mean measured value used was 1.669 Gy.			
36	18.55%	84.00%	99.80%
3	18.69%	52.33%	76.72%
**f.** Estimation at the point corresponding to Chamber 2: corresponding plan dose was 0.546 Gy. The QA phantom was re-setup between each measurement and additional 5% uncertainty was included. The mean measured value used was 0.471 Gy.			
36	0.00%	0.00%	0.00%
3	0.01%	0.06%	0.40%

As expected, at the location of Chamber 2, regardless of whether a conventional or SBRT treatment course was to be delivered, it was nearly impossible for the average treatment dose to be within 5% of the corresponding plan dose (Table 4b, d and f).

At the location corresponding to Chamber 1, for the conventional treatment course, even at the largest deviation (Table 4e), the average treatment dose was still almost certain to be within 5% of the plan dose (confidence level of 99.8%). With the deviation from the machine delivery alone, it was almost guaranteed that the treatment would be within 3% of the plan dose (Table 4a, the confidence level was 100%). If the re-setup uncertainty was added (Table 4c), that confidence level dropped to 94%. If the additional 5% clinical uncertainty was further taken into account (Table 4c), the confidence level to be within 3% decreased to 84%, indicating there was a need to reduce clinical uncertainty to ensure treatment quality. For the assumed SBRT treatment course, the uncertainty caused by the phantom re-setup alone brought the confidence levels of the 3% and 5% from 100% down to 65.58% and 90.16%, respectively (Table 4a and c). The additional 5% clinical uncertainty decreased the confidence levels even further to 52.33% and 76.72%, respectively. These results indicated that to ensure SBRT treatment quality as planned there is a need to apply more stringent requirement to minimize any source of uncertainties.

From Table 4a, c and e, it is interesting to note that with the same amount of treatment uncertainty the confidence levels were higher for the SBRT treatment course to achieve high level of treatment accuracy (e.g., within 1% of the plan dose) than the conventional treatment course. It can be explained by the fact that the standard deviation of the average delivery dose in a SBRT treatment course is larger than that of a conventional treatment course (according to Eqn. 11) and the larger standard deviation leads to a broader distribution which may be more likely to span over the plan dose.

## Discussion

This study is to establish a model taking the radiation delivery random errors into account. Even after taking the random errors into account, the expected measurement result may still differ from the corresponding planning quantity. This difference is likely caused by the systematic errors that originate from different sources, such as planning algorithm inaccuracy, machine calibration deviation, etc. These systematic errors can be potentially significant.

One of the major purposes of dosimetry measurements is to identify the expected value of the subject of measurement. For certain measurement procedures such as PSIMRT plan verification QA, this expected value of measurement may then be compared to a given value from the treatment plan. The expected value can only be quantified using measurement results, such as the mean and standard deviation, by using statistical concepts like probability, confidence level, and interval. Although a single measurement may provide a numerical value for the subject of measurement, its statistical relevance and significance is impossible to define. To obtain statistically meaningful results, at least two independent measurements must be performed.

From a statistical perspective, the number of measurements should not be predetermined. What should be predetermined are the desired confidence level and interval based on required dosimetric accuracy. The number of required measurements is then dependent on the measurement variance and the chosen confidence interval and level. According to the results presented in Results Section 3.1, it is obvious that smaller measurement deviations require a fewer number of required measurements.

Percent accuracy tolerances have been recommended for radiation treatment beam calibrations and PSIMRT plan verification QA [6,8,9]. During those procedures, the current practice is to take measurement(s) and compare the mean values to corresponding desired values (e.g., IMRT planned dose values or 1 cGy/MU in the case of machine calibration measurements). Decisions are then made based on whether the values are within tolerance. As demonstrated in the previous sections, this type of decision-making may be flawed since the confidence level of such accuracy is very dependent on the measured variance and number of measurements, and so should be evaluated based on not only simple comparison of the mean values to the given values but also measurement details such as measurement deviations and number of measurements. Moreover, it is anticipated that the doses delivered according to an IMRT treatment plan vary from fraction to fraction and exhibit a statistical distribution even if all machine components function within specifications. The standard deviation of this distribution may be influenced by several factors, such as machine delivery variation, patient setup variation, patient organ motion and body contour change, etc. On the other hand, IMRT treatment consists of a limited number of fractions, sometimes only a few fractions (e.g., Stereotactic Body Radiotherapy). The standard deviation of the expected average dose over a treatment course is not only dependent on the standard deviation of individual treatments but also highly dependent on the number of fractions (Eqn. 9). Thus, evaluation of a QA measurement outcome should also take into consideration the details of the treatment course for which the QA is performed. For example, as shown in Table 3c, after five QA measurements, the percent difference between the average QA result and the corresponding plan dose was less than 3% (-2.19%). Using the common practice of direct comparison with a preset tolerance of 3%, one would likely draw a conclusion that the QA result was acceptable. However, based on the statistical analysis, it was found that the confidence level was only 65% for the expected dose to be within 3% of the plan dose in an individual dose delivery, making it a little difficult to decide whether the plan was indeed acceptable for patient treatment. On the other hand, if the treatment course consisted of 36 fractions, the confidence level was 94% for the expected average treatment dose to be within 3% of the plan dose (Table 4c). In this case, the QAed plan could be deemed acceptable for this treatment course. Conversely, if the treatment course consisted of only 3 fractions, the confidence level was only 65.6% for the expected average dose to be within 3% of the plan dose over this hypo-fractionated course. In this later case, whether or not the QAed plan should be used for treatment might become questionable and action might be required to either modify the plan or adjust the beam delivery system to ensure treatment quality.

In a typical PSIMRT plan verification QA procedure, there is more than one point of interest that is evaluated by measurement and compared to a corresponding plan value. Thus, the decision making process is actually more difficult than the cases presented in this study. On the other hand, the basic principle still holds that a single measurement showing agreement or disagreement with the corresponding plan value cannot be used to draw a definitive conclusion in the pass or failure of the PSIMRT plan verification QA.

The purpose of PSIMRT plan verification QA is to verify the accuracy and precision of plan delivery. The current standard for PSIMRT plan verification QA verifies only the accuracy of plan delivery without providing statistical details. The ultimate goal of the plan QA is to ensure that the average dose delivered over a treatment course is within a desired tolerance with the plan. According to the proposed method, determining the accuracy of an IMRT plan requires multiple measurements and the information about the treatment course itself. The standard deviation in Eqns 10 and 11 should contain the contributions from various uncertainty sources, such as machine delivery, patient setup, anatomic motion and deformation, day-to-day machine variations, etc. Unfortunately, the only component that the conventional QA measurements can detect relatively accurately is the machine plan delivery variation. Therefore, a more reasonable way of evaluating a QA outcome may be as follows. First, a desired accuracy tolerance (e.g., the percent dose difference between plan and average delivery dose) with a specified confidence level is decided upon for the to-be-QAed treatment course. Second, a percent standard deviation (uncertainty) is estimated for the clinical patient treatment based on the patient anatomy study and motion evaluation (e.g., 4DCT for motion analysis), day-to-day machine stability, estimated patient setup variation, etc. Third, two QA measurements are performed and the results are analyzed using Eqns. 9–13. Fourth, if the subsequent confidence level does not meet the specified confidence level for the desired accuracy, additional QA measurements are conducted and the results are analyzed until no improvements in the confidence level are seen. Fifth, if the confidence level still does not meet the specified level, either the clinical patient treatment uncertainty needs to be further reduced or the plan needs to be revised.

In the analysis throughout the current study, measurement equipment uncertainty was not taken into account. If equipment random uncertainty (denoted as *σ*_*equip*_) is known, the measurement deviation for the subject of interest can be approximated as ${\bar{\sigma }}_{n}=\sqrt{{\bar{\sigma }}_{\mathrm{measurement}}^{2}-\sigma_{\mathrm{equip}}^{2}}$. If equipment is found to contribute to systematic errors, they should be identified and corrected for.

Dose distributions in conformal radiation treatments (e.g., IMRT and 3DCRT) can exhibit signification variations. Although the presented method is applicable to any point inside a phantom/patient, the derived statistical results most likely vary at different locations. Thus, a more comprehensive three dimensional approach is required to analyze the dose coverage inside a patient. This three dimensional analytical approach, taking many points into consideration, is beyond the scope of the current study and is subject to further investigation.

QA device is available now to simultaneously measure delivered doses at many different points. In principle, it is reasonable to utilize the measurement results at numerous points to derive the delivery variance, assuming that the dose measurement equipment is perfect. Unfortunately, the currently available measurement equipment has its own intrinsic limitations. Depending on the measurement region (e.g. high dose gradient regions vs low dose gradient regions), the variance introduced by the measurement equipment can be different at different locations. Careful analysis is required to utilize the measurement results at number locations for this purpose. On the other hand, the measurement results, obtained from many comparable patient QA measurements, can be useful to estimate the variance. The method and analysis presented in this study require two conditions: 1) measurements are independent and 2) the measurement results are normally distributed. The first condition is easily satisfied since one measurement does not affect the others, while the second condition is still an assumption, though generally accepted. If measurement of a subject of interest is proven to having something other than a normal distribution, the results and conclusions from the current study are not applicable.

The analysis presented above assumed that the difference in dose calculated for a QA phantom relative to the dose actually delivered by that machine to the phantom, is similar to the difference in dose calculated for a patient relative to the dose delivered to the same rigidly positioned patient by the same machine. This assumption is approximately true given that the phantom materials are similar to patient tissues. However, it needs to be again emphasized that there exist other errors, such as uncertainties in CT numbers, anatomical changes between simulation and treatment and during treatment, beam calibration variation, etc [18], which may lead to additional overall dose delivery uncertainty.

We acknowledge that although we believe our method to be scientifically sound it clearly will add to the workload of medical physicists and its practicality remains to be evaluated. Furthermore, in many centers, pre-treatment dosimetric verification is not carried out for every patient but only in a limited number of complicated cases. Therefore the feasibility of implementing the methods described in this work to assess PSIMRT QA results, although valid from a theoretical/methodological point of view, would have to be evaluated in the clinical scenario and perhaps combined with a population systematic and random errors based approach (van Herk, 2004) [23]. On the other hand, if the QA passing tolerance is established and delivery process uncertainty is established in a department, with the developed method, one QA measurement result should yield to a probability value for the QA to pass. If the probability value is higher than a certain acceptable level (e.g., 95%), no additional measurement is needed. The results of the current study were based on the assumptions that there were no human errors and that the user’s equipment was in good condition during measurements. If the measurement deviation is larger than usual, equipment malfunction must be ruled out. Rote adherence to this statistical method and approach without careful examination and analysis could lead to serious errors. It is also noticed that the some measurement data presented in this study exhibited certain non-randomness. It could be coincidence since all involved equipment were carefully evaluated and underwent adequate warm-up process before measurements.

## Conclusions

By acknowledging and considering the statistical nature of multi-fractional delivery of radiation treatment, we have established and demonstrated a quantitative methodology to evaluate the PSIMRT QA results. Both the statistical parameters associated with QA measurement procedures and the treatment course itself need to be taken into account to evaluate the QA outcome and to determine whether the plan is acceptable and whether additional measures should be taken to reduce treatment uncertainties. The result from a single QA measurement without statistical analysis can be misleading. When the required number of measurements is comparable to the planned number of fractions and the variance is unacceptably high, action must be taken to either modify the plan or adjust the beam delivery system.

## Competing interests

The authors declare that they have no competing interests.

## Authors’ contributions

NJY derived the mathematical equations; SBQ and MZ designed the experiments and collected data; SBQ, MZ, TC, LHK and NJY discussed the data collections and underlining physics and mathematics. SK and BGH provided clinical advices and guidance. All authors contributed, read and approved the final manuscript.
